# The Fundamental Need for Sleep in Neurocritical Care Units: Time for a Paradigm Shift

**DOI:** 10.3389/fneur.2021.637250

**Published:** 2021-06-17

**Authors:** Kislay Kishore, Michael D. Cusimano

**Affiliations:** Division of Neurosurgery, St. Michael's Hospital, University of Toronto, Toronto, ON, Canada

**Keywords:** sleep deprivation in neurocritical care, therapeutic sleep, frequency of neuromonitoring, sleep assessment in neurocritical care, need for sleep for recovery

## Abstract

Intensive neurological assessments in neurocritical care settings for unduly prolonged period result in profound sleep deprivation in those patients that confounds the true neurological status of these patients, and the mounting apprehension in providers can beget a vicious cycle of even more intensive neurological assessments resulting in further sleep deprivation from being constantly woken up to be “assessed.” This iatrogenic state drives these patients into deep sleep stages that impact spontaneous breathing trials, weaken immunity, and lead to unwarranted investigations and interventions. There is dwindling value of prolonged frequent neurochecks beyond the initial 24–48 h of an intracranial event. We insist that sleep must be considered on at least an equal par to other functions that are routinely assessed. We reason that therapeutic sleep must be allowed to these patients in suitable amounts especially beyond the first 36–48 h to achieve ideal and swift recovery. This merits a paradigm shift.

## Introduction

Sleep is a fundamentally integral function of all forms of life on this planet. The very fact that it comes at an expense of alertness and vulnerability to predators indicates that it must serve a physiologically indispensable purpose. Sleep deprivation is well known, commonly experienced, and results in a multitude of adverse effects in mental and physical wellbeing. The importance of sleep-promoting behavior as a fundamental component of wholesome wellbeing and recovery of patients is subsumed in neurosurgical or neurological patients where strict, timely neurological assessments are of critical value and patients' sleep often gets overlooked in the bargain.

Our thesis is that neurocritically ill patients in intensive care units require sleep and that depriving them of sleep can contribute to previously unrecognized significant morbidity and mortality in these patients. We highlight the need to safely balance the intensity of “neurochecks” while facilitating adequate sleep and physiological restoration in these patients. We argue that beyond the first 24–48 h, intensive monitoring can have more destructive effects than benefits and that patients need to be allowed to sleep if they are to recover efficiently and fully.

In this paper, we articulate the reasons why a paradigm shift in the way we manage neurocritically ill patients is required. We first contend that these patients often end up profoundly sleep deprived and to their serious peril. Sleep deprivation in these patients specifically confounds the true neurological status of these patients, and the mounting anxiety in providers can beget a vicious cycle of even more intensive neurological assessments resulting in further sleep deprivation from being constantly woken up to be “assessed.” This iatrogenic sleep-deprived state drives these patients into deep sleep stages that impair and confound neurological assessments and spontaneous breathing trials, impair immunity, and lead to unwarranted investigations like CT scans and even interventions such as anticonvulsants and ventricular cerebrospinal fluid (CSF) drainage.

Next, we review the pertinent literature that outlines the frequency of neurochecks and the diminishing value of frequent neurochecks beyond the first day or two in the neurocritical care unit.

In ending, we insist that sleep must be considered on *at least* an equal par to other functions, such as ventilator and renal function, which are routinely assessed daily on patients in neurocritical care units. We reason that therapeutic sleep must be allowed to these patients in appropriate amounts especially beyond the first 36–48 h to achieve optimal and swift recovery. Until we see clinical leaders support this position, patients entering neurocritical care units and being intensively monitored beyond 48 h will do so with the unnecessary added risks incurred by profound sleep deprivation. Restoring the sleep owed to them will pay therapeutic dividends of which we now inadvertently deprive the majority of our patients.

## Search Strategy

We used a conceptual review (1) format to better understand the contemporary concepts and themes relevant to this topic. Search criteria were based on Medline, Embase, Cochrane Database of Systematic Reviews, and Google Scholar using indexed words and keywords as detailed below. Common acronyms were searched in abstracts only. The following keywords “sleep deprivation in intensive care,” “sleep deprivation in neurological patients,” “sleep disturbances in ICU,” “sleep deprivation and neural plasticity,” “frequency of neurological assessments,” “neurointensive care monitoring,” and “glymphatic system” were used. We also used the search feature “similar articles” on PubMed, and the reference lists of relevant articles to identify additional sources. After deduplication, a total of 552 abstracts were scanned and relevant complete articles were reviewed and selected by the authors. The key concepts from these articles form the basis for this paper.

## Brief Overview of Normal Sleep Physiology

### The Vital Functions of Sleep

It was previously thought that sleep is a passive state of rest for the brain, but it is now well-established that brain functions continue throughout the sleep, albeit at a lower energy expenditure. Wakefulness is associated with intense and prolific synaptic activity and formation of synaptic long-term potentiations (LTPs) (2, 3). There is expenditure of energy by the excitatory neurotransmitter glutamate during awake state. Sleep results in downscaling of this heightened synaptic activity and promotes renormalization of synapses (4, 5), so that they are ready for another day of play.

Sleep also plays an important role in neural regeneration, repair, and plasticity (5). The theory of synaptic homeostasis and sleep-induced renormalization of synapses posits that synaptic potentiations during awake state are directly related to the slow wave activity during sleep (4). Slow-wave activity is a measure of “sleep need,” and longer periods of wakefulness incurs higher need to sleep (6). It has been found that higher LTP in a particular cortical area due to specific task while awake results in a higher slow-wave activity in the similar region of brain during the subsequent sleep (5). This sleep-mediated synaptic homeostasis is essential for conservation of cellular energy and optimal functioning of the brain. Thus, allowing neurocritically ill patients to sleep is not only physiological but also therapeutic.

In addition, sleep is also crucial for memory consolidation (7, 8). It has been shown that the synapses developed and activated during particular learning tasks are “reactivated” during sleep so as to consolidate the experiences gained. Furthermore, different stages of sleep have different roles in various types of memory formation. Slow-wave sleep is more crucial in consolidation of declarative memory, while rapid eye movement (REM) stage appears more important for procedural memory (9). However, these dual processes are not exclusive but instead represent a “double-step” phenomenon with both stages of sleep involved regardless of the elements of memory (10). Parallel synapses transfer the data from hippocampus to neocortex multiple times to etch a long-term memory during sleep (11). This can be simplified by imagining how repetitively drawn lines with a graphite pencil make it progressively darker and more difficult to erase. If we continuously awaken people, these lines never get made. It is therefore not surprising that neurodegenerative disorders like Alzheimer's disease, Parkinson's disease, and other dementia syndromes all have similar sleep disturbances characterized by exaggerated forms of naturally fragmented sleep patterns that are commonly observed in the elderly population (12).

### Glymphatic System

The glymphatic system is primarily active during sleep and is considered essential in promoting brain recovery and healing. This macroscopic waste clearance system of the brain, described as recently as 2012, is akin to the lymphatic system in the rest of the body. It is dependent on glial aquaporin-4 channels, hence the name “glymphatic.” The glymphatic system functions to provide convective movement (bulk flow; nearly equal rate of movement of small and larger molecules) of CSF along the periarterial spaces, and into the brain parenchyma by aquaporin-4 channels, which subsequently drive interstitial parenchymal fluid and macromolecules toward the perivenous space and ultimately *via* the jugular veins into cervical lymphatics, thereby cleansing the brain of unwanted macromolecules (13).

Cerebrospinal fluid influx into periarterial spaces is decreased by about 90% during awake state, and the interstitial space volume fraction is also 60% lesser during awake state. This is thought to be mediated by norepinephrine, which facilitates wakefulness and alertness, and is known to be a suppressor of glymphatic system by various mechanisms such as decreased interstitial space and reduced CSF production by choroid plexus. The glymphatic system is proposed to be useful in detoxifying the brain of protein aggregates such as β-amyloid, tau proteins, α-synuclein, and in the prevention of secondary neuronal damage due to accumulation of such molecules after traumatic brain injury. Clearance of brain interstitial solutes and wastes is one of the most important biological processes underlying the restorative function of sleep (14). The longer we deprive patients of this system function through frequent neuro-vital checks, the greater we risk accumulation of neurotoxins in the brain.

### The Normal Sleep Cycle

Sleep EEG has characterized normal sleep into a cycle of events transitioning from non-REM (NREM) stage to REM stage and over again (Table 1). The NREM stage constitutes almost 80% of the total sleep time and REM 20% (15, 16). A sleep cycle typically completes in about 90–110 min and occurs 4–5 times per night. It is imperative to realize that for there to be a restorative benefit, the entire sleep cycle must complete (17). Periodic interruptions result in staggered, non-restorative sleep. Preservation of this sleep *architecture* is more important than just a prolonged total sleep time as commonly occurs in pharmacologically induced/modified sedation (16).

**Table 1:** The normal sleep cycle.

NREM-N1	Transition between wakefulness and sleep
NREM-N2	Further decreasing awareness of surroundings, but still easily rousable by noise. Constitutes 45–55% of total sleep time.
NREM-N3 (slow wave sleep)	Deep sleep. Most difficult to arouse from. Most restorative stage. Growth hormone secretion peaks.
REM	Muscular atonia. Also has restorative benefits. Difficult to arouse but easier as compared to N3

It is commonly perceived that sedated patients in neuro-ICU are “sleeping,” and the assumption is that they are therefore adequately rested. However, most pharmacological agents used for sedation do not replicate natural sleep; total sleep time is increased but with altered sleep architecture. For example, drugs such as propofol, fentanyl, benzodiazepines, and morphine increase total sleep time but decrease the critical NREM-N3 and REM stages (18). Only dexmedetomidine (probably the closest to providing natural sleep) and haloperidol increase the NREM-N3 stage, although they too decrease the REM sleep (19, 20). It is clear that pharmacologically induced sleep does not have the same restorative benefits, and it is fallacious to presume those patients as adequately “rested.”

## Sleep Deprivation

The pathophysiological effects of sleep deprivation result from the composite lack of sleep and a period of extended wakefulness. Different neurochemical pathways mediate sleep and wakefulness in the brain, and the net effects of sleep deprivation are therefore greater than just lack of sleep (21). A concise summary of detrimental effects of sleep deprivation on various physiological systems is presented in Table 2.

**Table 2:** Pathophysiology of sleep deprivation on different systems.

Respiratory system (15, 22)	Sleep deprivation decreases intrinsic ventilatory drive to hypoxic/hypercapnic states due to decreased chemoreceptor sensitivity. Weakens inspiratory muscle strength. Negatively impacts ability to wean off the ventilator.
Cardiovascular system (23, 24)	Increases heart rate and blood pressure. Impairs the physiological “nocturnal dip” in BP. Increased catecholamine activity and sympathetic surge.
Immune system (16, 25–27)	Decreases extravasation of adapter T cells. Decreases the activity of (Natural Killer) NK cells by ~30%. Impairs normal sleep (NREM) induced heat dissipation and lowering of fever. (Sleep can therefore be understood as a “Protective reflex,” that's why ill patients tend to sleep more or demand more sleep.)
Metabolic and endocrine system (24, 28–31)	Linked to insulin resistance and diabetes mellitus. Persistently elevated levels of catabolic/immunosuppressive cortisol/adrenaline hormones And suppressed levels of Growth hormone and insulin (which are anabolic and immuno-facilitatory hormones)
Neurocognitive system (14, 17, 31–34)	Results in delirium and disturbed circadian rhythm. Increases risk of seizures, stroke, Alzheimer's disease, Headache, memory impairment, difficulty in focusing and concentration, irritability, impaired judgement, and decision making. Stress and anxiety disorders. Linked with addiction behavior. Impaired functioning of Glymphatic system.

### The Sleep-Deprived Brain

Functional MRI studies have shown that the default mode network (DMN) functions as the task-negative network in a wakeful rest state and gets “switched off” in the alert state to facilitate other executive networks to play. Sleep deprivation results in abnormal perseveration of DMN through alert state during periods of prolonged wakefulness, impairing the integrity between anterior and posterior nodes of DMN (35–38). It hampers the adaptive functional segregation of different brain networks and disrupts the functional network connectivity globally in the brain (21). This results in the attention deficit and impaired cognitive and executive functions seen in sleep deprivation. Sleep deprivation also affects dopamine signaling in the brain (39). This has been studied in relation to impaired reward processing and addiction behavior as well. Dopamine promotes wakefulness, and abnormally elevated dopamine levels due to prolonged wakefulness have bystander effects on the mesolimbic reward system. Sleep deprivation has been known to result in impulsivity, impaired reward–value estimation, and risk-taking behavior (21).

Irritability and delirium occur due to heightened sympathetic activity, which occurs both as a result of extended wakefulness requiring adrenergic drive to sustain and due to lack of the normal REM sleep-induced homeostatic dip in the central adrenergic activity (33, 40). Normally, cyclical cortisol secretion is at its lowest during sleep and highest at around 8 a.m. coinciding with wakefulness. Chronic sleep deprivation results in abnormally increased generalized sympathetic tone and is implicated in the development of hypertension, glucose intolerance, stress, and anxiety disorders.

**Figure 1:**
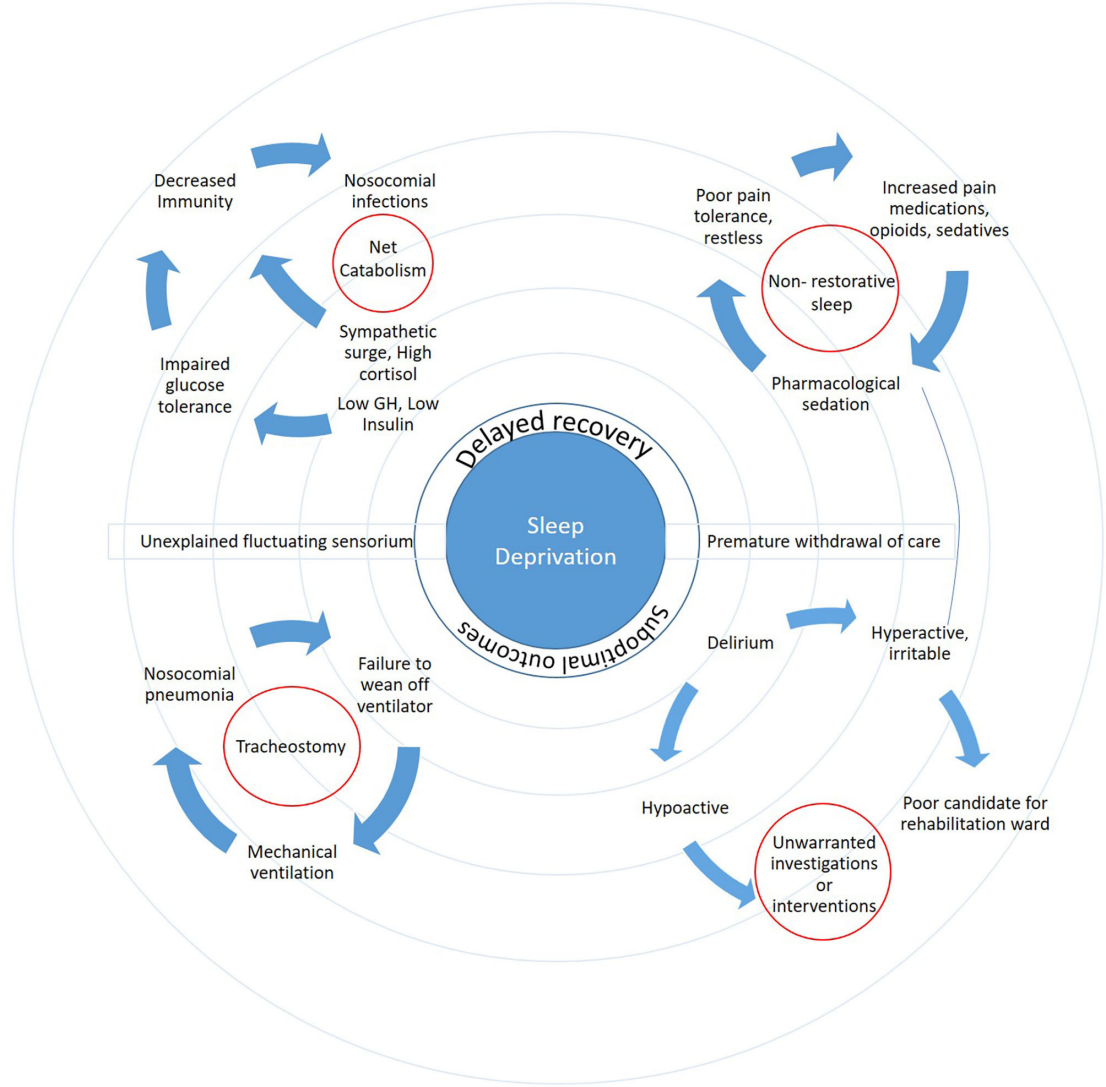
The ripple effect of sleep deprivation highlights the complex interactions and far-reaching consequences on patient outcomes.

Sleep deprivation results in increased cortical excitability in the brain. This is partially explained by lack of synaptic homeostasis and failure to downscale the glutamatergic activity, which is necessary to maintain forced prolonged wakefulness. Indeed, sleep loss has been shown to increase the propensity for seizures (32). A comprehensive overview of the vicious cycle of events triggered or propagated by sleep deprivation in neuro-patients is presented in Figure 1.

### Sleep “Debt” and Recovery Sleep

Both acute sleep deprivation and chronic sleep deprivation incur sleep “debt,” which can be quantified by increased delta and theta activities (slow-wave activity) in the frontal cortex (41). The usage of the word “debt” is highly literal here, and it truly indicates that the amount of recovery sleep required is not merely the same as the period of deprivation, but the “interest” incurred must also be repaid. The need for sleep increases with the length of wake period, and the longer the period of deprivation, the stronger the “sleep pressure” that develops. Sleep deprivation results in sleep pressure, which is an index of sleep debt (42). Recovery sleep has shorter sleep latency with longer periods of slow wave and REM sleep, so arousal can be tedious during the deep stages of recovery sleep (43). Lack of sleep recovery has direct impact on patients whose level of consciousness is constantly assessed and on patients who are placed on spontaneous breathing trials in preparation for extubation. In fact, research is now emerging that trying to extubate patients during periods of sleep debt is likely to fail (44).

## Balancing the Frequency of Neurochecks While Minimizing Sleep Deprivation

Frequent neurological assessments are crucial to detect early neurological deterioration and prompt timely intervention; this point is not in contention. However, we posit that prolonged intensive monitoring beyond the initial period is progressively counterproductive because of the accumulation of the negative effects of sleep deprivation in patients beyond the first 24–48 h.

Very few studies have examined the duration of intensive neurochecks in neurological/neurosurgical patients. A retrospective review of head trauma patients admitted in the ICU revealed 72% of patients were monitored with Q1h monitoring for almost 3 days. Of these, almost 40% had been classified as mild brain injury. Twenty percent of patients, the majority of whom had severe brain injury, were intensively monitored for longer than 4 days, whereas only 2% underwent surgical intervention beyond 48 h [both for chronic subdural hematoma (SDH) performed due to logistic issues or medical comorbidities other than neurological deterioration] (45).

In another study, a cohort of 132 patients with spontaneous intracerebral hematoma was observed with intensive hourly neurochecks for 5 days after admission to identify whether neurochecks serve as a biomarker of temporal profile in those patients. A timeline of 12 h after admission was identified as the period of maximum instability, with almost a 10 times reduction in possibility of change in Glasgow Coma Scale (GCS) points per hour after that (1.0 vs. 0.1). Even within the first 12 h, there was statistically significant diminution of yield in every subsequent 3-h block (46).

A prospective observational study of a heterogeneous cohort of neurosurgical patients showed that 63% of GCS decline occurred within the first 48 h of ICU admission, and of the remaining events that were detected by continued intensive neurochecks, 78% either showed no deterioration on imaging or no other attributed cause could be identified. Instead, they were thought to be due to delirium caused by sleep deprivation (47).

The chronological profile of traumatic intracerebral contusion/hematoma has also been described in an earlier study and revealed its evolution was complete in 100% of 48 subjects under study within the first 24 h after trauma, of which 80% had evolved within the first 12 h (48).

Taken together, these studies reveal that in most situations, intense Q1h neurochecks may be redundant beyond the first 24 h after an intracranial event but may sometimes be extended up to 48 h depending on clinical judgment. Prolonged periods of sleep deprivation negatively impact the “true” neurological status that patients are being assessed for and potentially provoke unwarranted and potentially harmful intervention. Subsequently, well-spaced out assessments would allow patients more restorative and therapeutic sleep.

A particular example where a rational and tempered approach that balances the risks and benefits of frequent neurochecks with the risks and benefits of sleep deprivation is in the case of patients with subarachnoid hemorrhage (SAH). These patients, like all others, should have an approach that recognizes the benefits of more frequent neurological checks during periods of highest risk of sustaining permanent neurological deficit. The risk that a patient has for delayed ischemic neurological deficit (DIND) after SAH has been the subject of many papers, and these papers uniformly report severity of the initial bleed as a risk factor for DIND. One such practical protocol based on the severity of SAH, in terms of World Federation of Neurological Surgeons (WFNS) grade and modified Fisher grade, is called VASOGRADE, and it color-codes these patients into the patients with the highest risk of DIND into the red zone and the lowest risk group into the green zone (49). One algorithm, albeit without empirical support, suggests Q4h neuro monitoring in green zone patients, Q2h in yellow zone patients, and Q1h monitoring in red zone patients (50). However, the duration for which this should be continued is not clearly stated. The astute clinician will look for potential signs of impending vasospasm such as fever, which may herald the onset of vasospasm in the absence of an infectious focus (51). For SAH patients, particularly those with higher-grade SAH, Q1h neurochecks for 10 days would have patients sustain systemic repercussions from tremendous sleep deficits so many centers now utilize multimodality advanced monitoring [brain tissue oxygen PbO_2_, lactate–pyruvate ratio, intracranial pressure (ICP)], which provide useful actionable information to detect ischemic risk in up to 20% instances. This approach must still however be balanced with “neurological wake-up tests” in those who are pharmacologically sedated (52).

In a similar fashion, one could argue for a rationalized approach in patients with large infarcts and intracerebral bleeds in that a balanced application of frequent checks and allowing sleep be worked around times of maximal herniation risks peaking usually at around days 3–5 post event (53). A blanket and automated system will likely not work for everyone; cases need to be individualized.

We emphasize that a rational, thoughtful, and tempered approach be taken in assessing the dynamics of the context of individual neurologically ill patients. It is clear that an individualized approach is required. Our insights and recommendations must not be construed as surmounting the judgment of an experienced clinician but as a beacon to help clinicians harness the therapeutic effects of sleep and at the same time always being aware of the balance of the detrimental effects of sleep deprivation when instituting management plans that affect sleep. Future research will help identify those patients in whom the balance should lean more toward frequent checks and others where the balance should be to allow sleep to occur. We remain convinced that all clinicians should always keep in mind that restorative sleep should be at the core of a therapeutic approach to the patient's recovery.

## The Imperative for Change: Reintroducing Sleep into Neurological Care

The fundamental role that sleep plays in a multitude of bodily functions, in all animals, including humans, is neglected by modern medicine. This is particularly true if one walks into any neurocritical care unit. Here, patients are often constantly “monitored” hourly with GCS scores, ICP monitors, arterial lines, Foley catheters, and a multitude of scans and other interventions in the name of the benefit to the patient.

Change is occurring in general critical care settings. There is an increased awareness in non-neuro-patient settings to create environments conducive to sleep. These include practical approaches at modifying ambient noise levels by providing earplugs or white noise, simulation of ambient lighting to closely mimic day–night rhythm, and clustering of patient care activities such as drug administration, blood sampling, and meal timings (54). However, sometimes, unscheduled “out of hours” investigations/scans are done to accommodate staff convenience (i.e., so results are available at rounds) and should be discouraged.

From a neurological perspective, it is abundantly clear that an important cause of fluctuating neurological status in neuro-patients may be unrecognized sleep deprivation. In addition, it is also obvious that the reparative, immune-facilitatory, and anabolic functions are required in patients with critical neurological illnesses. Addressing ways to balance these effects with the need to “monitor” patients will pay dividends to patient outcomes. Ultimately, this will require a broad culture change in neurocritical care areas and among all those involved in the care of these patients.

The most important first step to changing practice is addressing the lack of awareness pertaining to the magnitude of problem and the solutions that exist. Healthcare provider anxiety contributes to the frequency of neurochecks for prolonged periods of time, and there is an inherent resistance among staff to diminish the frequency of these neurochecks. We have reviewed the evidence that continued hourly neuro-monitoring beyond the first 24 or 48 h rarely leads to actionable intervention and can have deleterious effects that increase over time.

Instituted timely de-escalation of the frequency of neurochecks must be built into care pathways and routines so that the “returns” from continuing frequent checks is mandated on a daily basis. Such an approach could have additional beneficial effects such as lessening the need for frequent sedation pauses (which may in fact be detrimental in patients with traumatic brain injury and elevated ICPs) and subsequently lessen the burden on nurses.

To accomplish this, we recommend building a “sleep review” into daily routines of all caregivers who look after critically ill patients. This sleep review would be one of the items that trainees would be taught to incorporate into their daily physiological assessments of patients alongside assessments of other vital signs, respiratory, cardiac, neurological, renal, deep vein thrombosis prophylaxis, and other functions. Completion of these frequent “sleep reviews” should become a routine assessment, consciously checked and managed on a *daily basis* in every neurocritical care area, as well as a metric used in assessing the quality of care in neurocritical care areas. Making the assessment of sleep a part of the routine and mandatory assessments alongside respiratory, cardiac, renal, and neurological status will not only avoid the detrimental effects of sleep deprivation but allow patients to experience sleep's therapeutic effects.

An examination of the hierarchies in the care of critically ill patients alongside ensuring those “in charge” work to institute change is also important. Senior neurological specialists, intensivists, and nursing leaders should be encouraged to convey to more junior colleagues the importance and impact of scaled back frequency of neurochecks beyond the first 24–48 h. Having all members of a care team function with the same point of view regarding the de-escalation of monitoring intensity would mitigate anxiety and fear of admonishment for “not being vigilant enough.” A close collaboration between the neurosurgeon, neurologist, intensivist, nurses, and other members of the critical care team is essential to achieving this change.

## Conclusion

Sleep must be considered on at least an equal par to other functions, such as ventilator and renal function, which are routinely assessed daily on patients in neurocritical care units. We reason that therapeutic sleep must be allowed to these patients in suitable amounts especially beyond the first 24–48 h to achieve ideal and swift recovery. Sleep hygiene should be routinely “assessed” like any other parameter in neuro-ICUs. This calls for a paradigm shift among the clinical leaders to enforce such a change in practice.

Addressing sleep deprivation in neurocritically ill patients is in its infancy. Future research will uncover many unanswered questions. Eventually, working to mitigate these adversities for our patients will also shed light on our own practices around sleep and improve the health of everyone in neurocritical care areas.

## Data Availability Statement

The original contributions presented in the study are included in the article/supplementary material, further inquiries can be directed to the corresponding author/s.

## Author Contributions

All authors listed have made substantial, direct and intellectual contribution to the work, and approved it for publication.

## Conflict of Interest

The authors declare that the research was conducted in the absence of any commercial or financial relationships that could be construed as a potential conflict of interest.
